# Interrelationships Between Self-Injury Addiction, Traumatic Experiences, and Rumination Among Adolescents With Non-Suicidal Self-Injury: A Network Analysis

**DOI:** 10.31083/AP44340

**Published:** 2026-02-03

**Authors:** Shijian Wang, Lin Zhao, Doudou Zheng, Linghua Kong, Jingya Li, Ying Yang

**Affiliations:** ^1^Shandong Mental Health Center, Shandong University, 250014 Jinan, Shandong, China; ^2^School of Mental Health, Jining Medical University, 272013 Jining, Shandong, China; ^3^School of Nursing and Rehabilitation, Cheeloo College of Medicine, Shandong University, 250012 Jinan, Shandong, China

**Keywords:** adolescents, NSSI addiction, trauma, rumination, network analysis

## Abstract

**Background:**

Non-suicidal self-injury (NSSI) is on the rise in adolescent populations and its addictive profile, marked by frequent repetition, severe damage, and higher suicide risk, has raised broad concern. Childhood trauma is a key influencing factor that enhances emotional sensitivity and increases susceptibility to NSSI. Rumination, characterized by persistent negative thoughts, may mediate this association by amplifying emotional distress, as suggested by the emotional cascade model. Guided by the Interaction of Person–Affect–Cognition–Execution (I-PACE) model, this study employed network analysis to investigate the interactive associations among childhood trauma, rumination, and NSSI addiction in adolescents, aiming to identify core and bridge symptoms.

**Methods:**

We enrolled 1169 adolescents with NSSI and collected data using demographic questionnaires along with the Ottawa Self-Injury Inventory, Childhood Trauma Questionnaire, and Ruminative Responses Scale. Undirected network and Bayesian network analyses were applied to examine the complex associations among symptoms and mediation analysis was performed guided by the directed acyclic graph structure.

**Results:**

By integrating both directed and undirected network models, symptom rumination and emotional abuse were identified as central nodes influencing the addictive nature of self-injury. Mediation analysis supported the pathway suggested by the directed acyclic graph (DAG), showing that symptom rumination mediated the relationship between emotional abuse and NSSI addiction. Network comparison further indicated that this link between self-injury addiction and symptom rumination was stronger in the addiction group than in the non-addiction group.

**Conclusion:**

In Chinese adolescents, timely identification and intervention targeting rumination on emotionally abusive experiences may reduce the onset and persistence of NSSI addiction.

## Main Points

(1) Central Role of Emotional Abuse and Symptom Rumination: Network analysis identified emotional abuse and symptom rumination as key nodes influencing non-suicidal self-injury (NSSI) addiction in adolescents, acting as critical drivers in the trauma-rumination-addiction network.

(2) Mediation Pathway: Mediation analysis confirmed that symptom rumination mediates the relationship between emotional abuse and NSSI addiction, with rumination accounting for 40% of the total effect, highlighting a significant pathway for intervention.

(3) Stronger Association in Addiction Group: Comparative network analysis showed a stronger connection between symptom rumination and NSSI addiction in the addiction group compared to the non-addiction group, emphasizing rumination’s role in addictive NSSI behaviors.

(4) Intervention Implications: Targeting symptom rumination, particularly in the context of emotional abuse, through interventions like rumination-focused cognitive behavioral therapy (RF-CBT) may reduce the risk and persistence of NSSI addiction in adolescents.

(5) Innovative Methodological Approach: The study innovatively combined undirected network analysis and Bayesian network analysis with mediation analysis to elucidate the complex relationships among childhood trauma, rumination, and NSSI addiction, providing a robust framework for identifying key symptoms and causal pathways.

## 1. Introduction

Non-suicidal self-injury (NSSI) is defined as deliberate self-harm without suicidal intent and has become increasingly common in adolescents, with lifetime prevalence in China reported as high as 24.7% [1, 2]. Adolescents may resort to NSSI when facing negative emotions or stress, gaining short-term relief; yet this relief tends to reinforce repetition in similar contexts, fostering addictive tendencies [3]. An existing study has demonstrated that addictive NSSI involves increased frequency, more extensive injury sites, and greater injury severity, potentially leading to severe physical trauma, functional impairment, and elevated suicide risk [4]. This is clinically relevant, as conceptualizing NSSI as an addictive behavior may inform the development of targeted interventions [5].

NSSI arises from multifaceted influences, including biological, psychological, social, and cultural contexts, with childhood trauma being a particularly salient factor [6]. Childhood trauma denotes severe adverse experiences during early life [7]. Prior evidence indicates that such experiences heighten vulnerability to negative events, leading to sustained emotional distress and impaired social adaptation, which in turn elevate the likelihood of NSSI [8]. A recent study involving Chinese adolescents revealed significantly higher prevalence rates of NSSI among those with histories of emotional abuse (58.9%), physical abuse (46.3%), and sexual abuse (62%) compared to their non-abused counterparts [9]. The strength of the association between childhood trauma severity and NSSI behaviors surpasses that of other conventional risk factors, further underscoring the critical role childhood trauma plays in NSSI development [10].

Rumination is considered a key cognitive mechanism connecting childhood trauma with NSSI, involving repetitive focus on negative experiences and sustained attention to distressing emotions [11]. Selby’s emotional cascade model indicates that rumination heightens negative affect and establishes a recursive cycle, increasing the likelihood of NSSI [12]. This cycle produces escalating emotional distress, driving individuals to use NSSI as a temporary strategy to shift attention and interrupt the negative feedback loop [13]. Thus, rumination may function as a key mediator linking childhood trauma to NSSI, thereby increasing adolescents’ susceptibility to addiction-like self-injury patterns [14].

While connections between NSSI, trauma, and rumination have been studied, addiction-related aspects are understudied and no holistic analysis of their interactions is available. The Interaction of Person–Affect–Cognition–Execution (I-PACE) model serves as an integrative perspective for interpreting how addictive behaviors develop and are sustained [15]. According to network theory, psychopathological phenomena arise not from a single latent factor but from dynamically interacting symptoms that constitute a network [16]. Network analysis (NA), as an emerging data-driven method, has increasingly been utilized in recent years to elucidate complex associations among psychological symptoms, visually representing the roles and interconnections of core and bridge symptoms within symptom networks [17]. Through mapping core nodes and critical links among childhood trauma, rumination, and NSSI addiction, network analysis helps pinpoint intervention priorities and provides a solid theoretical and empirical basis for guiding prevention and clinical practice in adolescents. This study, therefore, employs network analytic methods to examine how childhood trauma, rumination, and NSSI addiction are interrelated in adolescent populations.

## 2. Methods

### 2.1 Participants

Between June and December 2024, adolescents aged 10–19 years were recruited through convenience sampling from the outpatient department of a tertiary psychiatric hospital in Shandong Province. Informed consent was obtained from both participants and their guardians, and questionnaires were administered via Wenjuanxing (https://www.wjx.cn/) . Socioeconomic status and parenting style were assessed by adolescents’ subjective self-report using the response options provided in the structured questionnaire (e.g., ‘superior, good, average, poor’ for socioeconomic status). Inclusion criteria were as follows: (1) age between 10 and 19 years at the time of enrollment (based on the WHO definition of adolescence); (2) the participant and their legal guardian provided informed consent after fully understanding the study procedures; and (3) adequate comprehension of the questionnaire content. Exclusion criteria included the presence of language barriers, limited reading ability, cognitive impairment, or acute psychiatric illness that would interfere with the completion of study assessments.

### 2.2 Measures

#### 2.2.1 Childhood Trauma Questionnaire (CTQ)

Bernstein *et al*. [18] created this questionnaire to measure traumatic experiences occurring during childhood. The questionnaire contains 28 items, with 25 assessing its core dimensions. Each item is rated on a 5-point Likert scale ranging from 1 (‘never’) to 5 (‘very often’). The questionnaire covers five trauma domains (emotional, sexual, and physical abuse; emotional and physical neglect), with subscale scores of 5–25 and a total score of 25–125. The Chinese version has proven reliable and valid [19]. This scale achieved a Cronbach’s alpha of 0.733, indicating acceptable internal consistency.

#### 2.2.2 Ruminative Responses Scale (RRS)

The RRS is a self-report questionnaire widely employed to assess rumination [20]. The scale contains 22 items across three domains—Symptom Rumination, Brooding, and Reflection—rated on a 4-point Likert scale. Higher scores indicate a stronger rumination tendency. The Chinese RRS has been validated in earlier studies, demonstrating reliable and valid psychometric properties [21]. The scale demonstrated excellent internal consistency, with a Cronbach’s alpha of 0.95.

#### 2.2.3 NSSI Addiction

To assess addiction-related characteristics, we used the Ottawa Self-Injury Inventory (OSI), which has been shown to possess satisfactory psychometric properties [4]. In this study, it served to assess addiction traits across seven items. Greater total scores indicate stronger addiction traits. Following the OSI scoring manual and prior validation work, each addictive-feature item was dichotomized at ≥2 (‘meets criterion’), and NSSI was classified as exhibiting addictive features when ≥3 of the 7 criteria were met. Accordingly, adolescents were categorized into addictive and non-addictive NSSI groups. The scale demonstrated a Cronbach’s alpha of 0.92, indicating excellent internal consistency.

### 2.3 Statistical Analysis

Use SPSS 26.0 (IBM Corp., Armonk, NY, USA) for descriptive analysis and mediation analysis, and use R version 4.4.2 (R Foundation for Statistical Computing, Vienna, Austria) for network analysis. Before network analysis, descriptive statistics were computed for both the addictive and non-addictive groups. Normality was assessed using the Kolmogorov–Smirnov and Shapiro–Wilk tests (all *p* < 0.001), with Q–Q plots, boxplots, and stem-and-leaf plots further confirming deviations, particularly at the distribution tails. Continuous variables with skewed distributions were expressed as medians (P25, P75), and group comparisons were assessed using the Mann–Whitney U test.

#### 2.3.1 Network Estimation

The psychopathological symptom network architecture was analyzed using validated R packages bootnet package (version 1.6, Sacha Epskamp; Comprehensive R Archive Network [CRAN], Vienna, Austria; https://github.com/SachaEpskamp/bootnet) and qgraph package (version 1.9.8, Sacha Epskamp; Comprehensive R Archive Network [CRAN]; https://github.com/SachaEpskamp/qgraph). Nodes reflected raw dimension scores, and a gaussian graphical model (GGM)-based partial correlation network was constructed, including trauma (5 nodes), rumination (3 nodes), and NSSI addiction (1 node). We employed graphical LASSO (gLASSO) regularization combined with Extended Bayesian Information Criterion (EBIC, γ = 0.5) to achieve an optimal trade-off between model fit and sparsity by shrinking trivial edge weights toward zero [22].

Network visualization was generated using the Fruchterman–Reingold algorithm, which places strongly connected nodes toward the center and weaker ones at the margins [22]. To quantify the importance of nodes, we adopted Expected Impact (EI) centrality-a weighted metric that considers the weights of positive and negative edges. This metric has higher reliability in psychological networks than traditional centrality indices (strength, closeness, and betweenness) [23, 24]. Bridge expected influence (BEI) was calculated to detect key transdiagnostic nodes with the highest cross-community connectivity, which may represent activation pathways underlying comorbid symptoms.

#### 2.3.2 Estimating Network Accuracy and Stability

Network stability was tested through case-dropping bootstrapping, and the Correlation-Stability Coefficient (CS-coefficient) was computed as advised in prior study. A CS-coefficient exceeding 0.25 was considered to represent moderate stability, whereas values above 0.5 denoted strong stability of node indices [22]. Accuracy was tested by non-parametric bootstrapping with 95% CIs, where narrower intervals implied better precision, and bootstrapped tests were used to compare edge weights and centrality indices.

#### 2.3.3 Bayesian Network (BN)

In accordance with the I-PACE model, which conceptualizes early life experiences as upstream vulnerability factors that shape later cognitive and affective processing, we imposed a theoretical constraint on the Bayesian network structure by fixing childhood emotional abuse as an exogenous node. Using the bnlearn hill-climbing algorithm, we estimated a Bayesian network and produced a directed acyclic graph (DAG) reflecting conditional dependencies [25]. To assess the robustness of the network structure, we conducted bootstrapping with 1000 resamples. Edges retained in the final averaged network appeared in at least 85% of the iterations with consistent directionality in over 50%. The network was rendered through the Rgraphviz 2.50.0 (Bioconductor Project, Seattle, WA, USA).

#### 2.3.4 Network Comparison Test

The Network Comparison Test (NCT) 2.2.2 (CRAN, Amsterdam, Netherlands) was used to evaluate differences in network characteristics between the addicted and non-addicted groups. Specifically, we tested network invariance by comparing the strongest edge, assessed global strength through the sum of all edge weights, and examined edge invariance by evaluating differences in individual connections between groups [26]. Edge-level differences were corrected for multiple comparisons with the Holm–Bonferroni method.

#### 2.3.5 Network Models Adjusted for Sex as a Covariate

Given known sex-related correlates of adolescent NSSI and the sex differences in our sample [27], network models were constructed with sex as a covariate. In order to test potential sex effects on the network, we reconstructed the NSSI, addictive, and non-addictive group networks while adjusting for sex. For each model, we compared original and sex-adjusted networks by correlating their edge-weight matrices.

#### 2.3.6 Mediation Analysis

To further validate the joint central nodes identified in both the undirected network and the Bayesian network, and to explore the underlying mechanism of the significant positive association between emotional abuse and addictive NSSI, symptom rumination was introduced as a mediating variable within a structural equation model. Mediation was examined with the PROCESS macro (Model 4) in SPSS. Table 1 shows that gender, grade, only-child status, parenting style, and device use differed significantly between the two NSSI groups. To test the mediating effect of M on the X–Y relationship, we applied the bootstrap procedure suggested by Hayes.

**Table 1.** **Characteristics of the study population by NSSI status N (%)**.

Variables		Non addictive	Addictive	Total	χ^2^	*p*
Sex						
	Male	170 (29.8)	123 (20.5)	293 (25.1)	13.422	<0.001
	Female	400 (70.2)	476 (79.5)	876 (74.9)		
Grade						
	Primary	21 (3.7)	24 (4.0)	45 (3.8)	28.753	<0.001
	Junior	239 (41.9)	342 (57.1)	581 (49.7)		
	Senior	288 (50.5)	215 (35.9)	503 (43.0)		
	University	22 (3.9)	18 (3.0)	40 (3.4)		
Parents’ marital status						
	First Marriage	495 (86.8)	491 (82.0)	986 (84.3)	7.233	0.065
	Divorced	38 (6.7)	49 (8.2)	87 (7.4)		
	One Deceased	11 (1.9)	11 (1.8)	22 (1.9)		
	Remarried	26 (4.6)	48 (8.0)	74 (6.3)		
Socioeconomic status						
	Superior	9 (1.6)	10 (1.7)	19 (1.6)	1.124	0.771
	Good	192 (33.7)	212 (35.4)	404 (34.6)		
	Average	333 (58.4)	333 (55.6)	666 (57.0)		
	Poor	36 (6.3)	44 (7.3)	80 (6.8)		
Only child						
	Yes	142 (24.9)	195 (32.6)	337 (28.8)	8.313	0.004
	No	428 (75.1)	404 (67.4)	832 (71.2)		
Parenting style						
	Authoritarian	99 (17.4)	95 (15.9)	194 (16.6)	18.804	0.001
	Neglectful	79 (13.9)	116 (19.4)	195 (16.7)		
	Authoritative	127 (22.3)	171 (28.5)	298 (25.5)		
	Permissive	37 (6.5)	38 (6.3)	75 (6.4)		
	Others (Democratic, Encouraging, etc.)	228 (40.0)	179 (29.9)	407 (34.8)		
Use of personal electronic equipment						
	Never	1 (0.2)	4 (0.7)	5 (0.4)	10.555	0.032
	Occasionally	27 (4.7)	28 (4.7)	55 (4.7)		
	Sometimes	86 (15.1)	76 (12.7)	162 (13.9)		
	Frequently	231 (40.5)	205 (34.2)	436 (37.3)		
	Daily	225 (39.5)	286 (47.7)	511 (43.7)		

Note: NSSI, non-suicidal self-injury.

## 3. Results

### 3.1 Sample Characteristics

We recruited 1681 adolescents aged 10–19 years from mental health outpatient services. Among the participants, 1169 (69.5%) reported self-harm behaviors, with males accounting for 25.1% of this subgroup. Table 1 presents the demographic characteristics of 1169 adolescents aged 10–19 years with self-injurious behaviors attending the psychiatric outpatient clinic. Among the participants, 599 individuals (51.2%) were classified into the addiction group. Significant differences were observed between the addiction groups in terms of gender, grade level, only-child status, parenting style, and the use of personal electronic devices (*p* < 0.05). In this sample, 78.6% of adolescents with self-injurious behaviors reported at least one type of childhood trauma, and this rate increased to 83.7% within the self-injury addiction group. Table 2 shows that the addiction group scored significantly higher than the non-addiction group across all dimensions of rumination and trauma (*p* < 0.05).

**Table 2.** **Non-parametric test for scale dimension scores across NSSI addiction groups (Med, P25, P75)**.

Variables		Non addictive	Addictive	Total	Z	*p*
RRS						
	Symptom rumination	31 (25, 37)	38 (32, 44)	35 (28, 41)	–12.448	<0.001
	Brooding	14 (11, 17)	16 (14, 19)	15 (12, 18)	–9.495	<0.001
	Reflective pondering	11 (9, 14)	14 (11, 16)	13 (10, 15)	–8.642	<0.001
CTQ						
	Emotional neglect	15 (11, 19)	17 (13, 21)	16 (12, 20)	–5.022	<0.001
	Emotional abuse	9 (7, 12)	12 (9, 16)	11 (8, 15)	–9.459	<0.001
	Physical neglect	9 (7, 11)	11 (8, 13)	10 (7, 12)	–6.730	<0.001
	Physical abuse	5 (5, 7)	6 (5, 9)	5 (5, 8)	–5.761	<0.001
	Sexual abuse	5 (5, 5)	5 (5, 6)	5 (5, 5)	–4.632	<0.001

Note: Med, median; RRS, Ruminative Responses Scale; CTQ, Childhood Trauma Questionnaire.

### 3.2 Network Analysis

#### 3.2.1 Network Estimation and Visualization

**Fig. 1.**
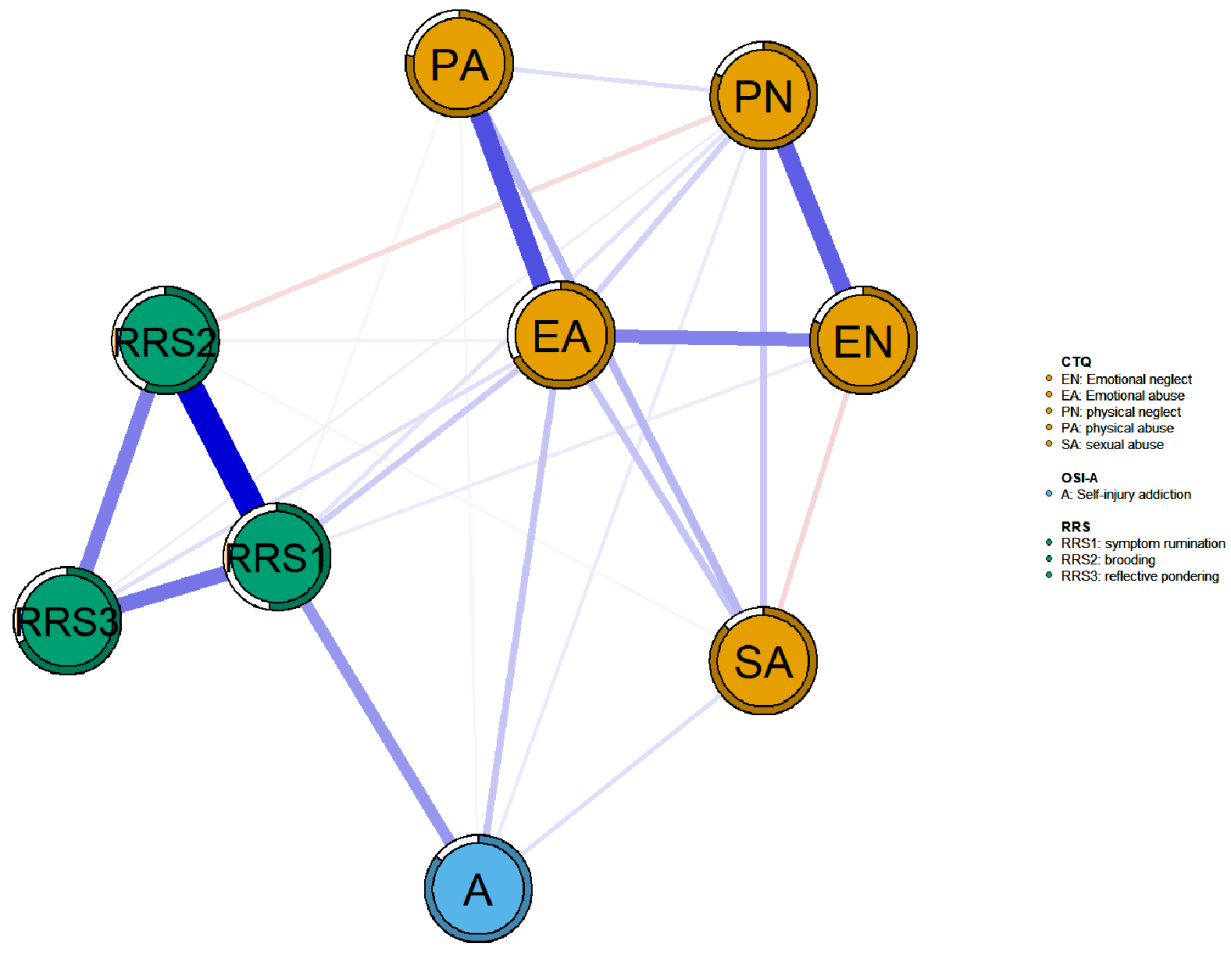
**Symptom networks of NSSI adolescent self-injury addiction, trauma, and rumination**. In the network, blue nodes denote self-injury addiction, green nodes indicate rumination, and orange nodes reflect trauma. Blue edges represent positive correlations, red edges negative ones, and line thickness corresponds to the strength of associations. The circular rings around nodes indicate node predictability (R^2^), representing the proportion of variance in each node explained by all other connected nodes. A, self-harm addiction; EN, emotional neglect; EA, emotional abuse; PN, physical neglect; PA, physical abuse; SA, sexual abuse; OSI-A, Ottawa Self-Injury Inventory-Addictive Features.

**Fig. 2.**
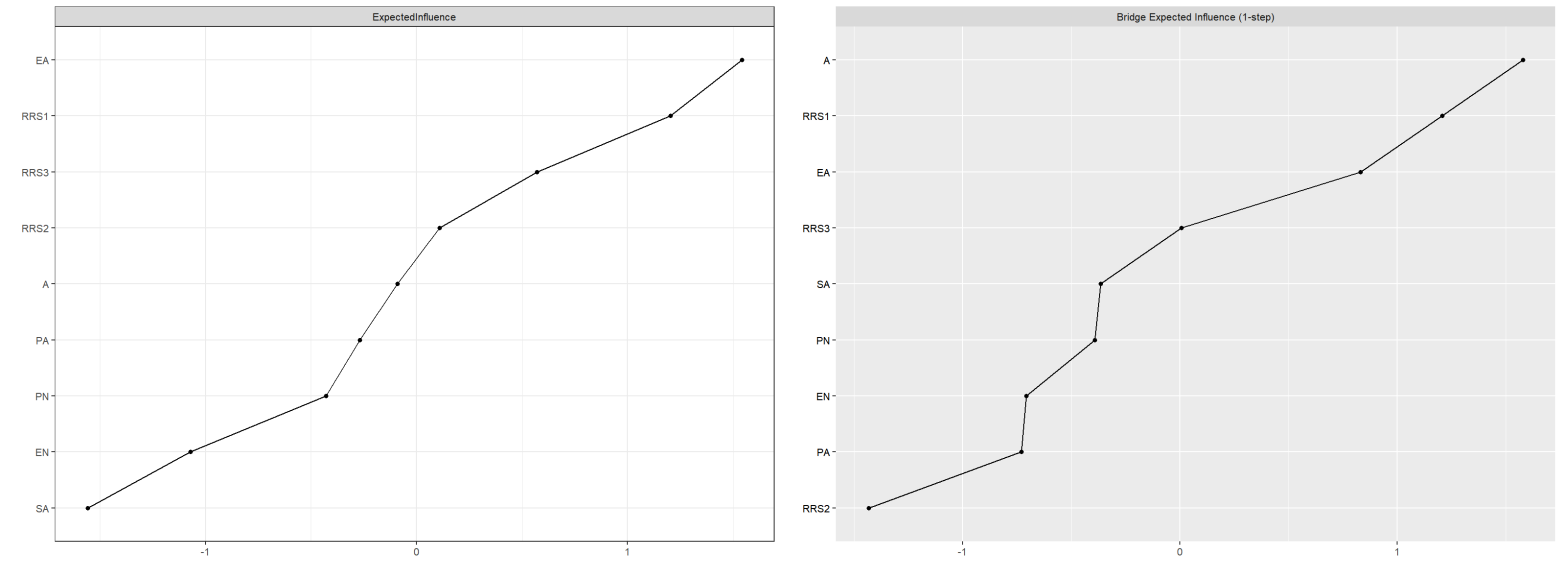
**Standardized EI and BEI for each node in the network**. A, self-harm addiction; RRS1, symptom rumination; RRS2, brooding; RRS3, reflective pondering.

Fig. 1 illustrates the trauma–rumination–self-harm addiction network, comprising 30 non-zero edges (83.3% of 36) with an average weight of 0.10. Within the rumination cluster, the most robust association was observed between RRS1 and RRS2 (weight = 0.59), whereas in the childhood trauma cluster, the strongest link was between emotional abuse (EA) and physical abuse (PA) (weight = 0.41). Across the self-harm addiction and other clusters, RRS1 showed the closest tie with addiction (weight = 0.23), followed by EA (weight = 0.13). A full list of edge weights is presented in **Supplementary Table 1**. Centrality and bridge centrality analyses highlighted symptom rumination and emotional abuse as pivotal nodes, given their extensive connections to other nodes and their potential to activate different communities. The centrality metrics of the network are illustrated in Fig. 2, with detailed numerical values provided in **Supplementary Table 2**.

#### 3.2.2 Network Stability and Accuracy

**Fig. 3.**
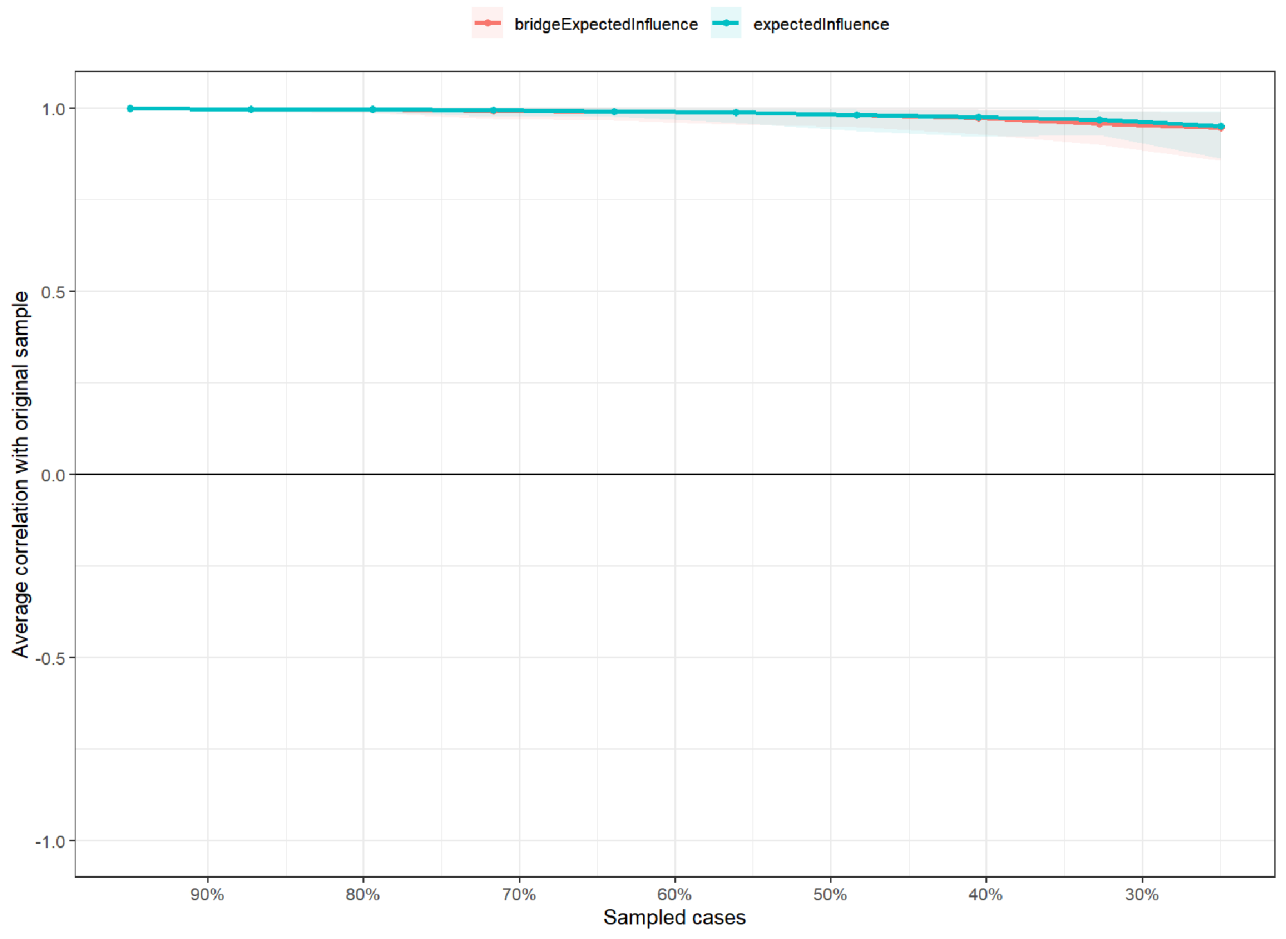
**Stability of centrality and bridge centrality indices (CS-C)**. Correlation stability coefficients (CS-coefficient) assessed the robustness of centrality indices using case-dropping subset bootstrap. The CS-C indicates the maximum proportion of cases that can be removed while maintaining, with 95% probability, a correlation ≥0.70 between original and subset centrality estimates. In this study, both indices showed high stability (CS-C = 0.75).

As illustrated in Fig. 3, the network demonstrated high stability, with CS coefficients of 0.75 for both EI and BEI. Bootstrapped 95% confidence intervals (**Supplementary Fig. 1**) confirmed the robustness of edge, EI, and bridge EI estimates. Moreover, bootstrap difference tests (**Supplementary Figs. 2,3**) indicated that most edges and EI values were significantly distinct.

#### 3.2.3 Probabilistic Causal Structure: DAG Analysis

**Fig. 4.**
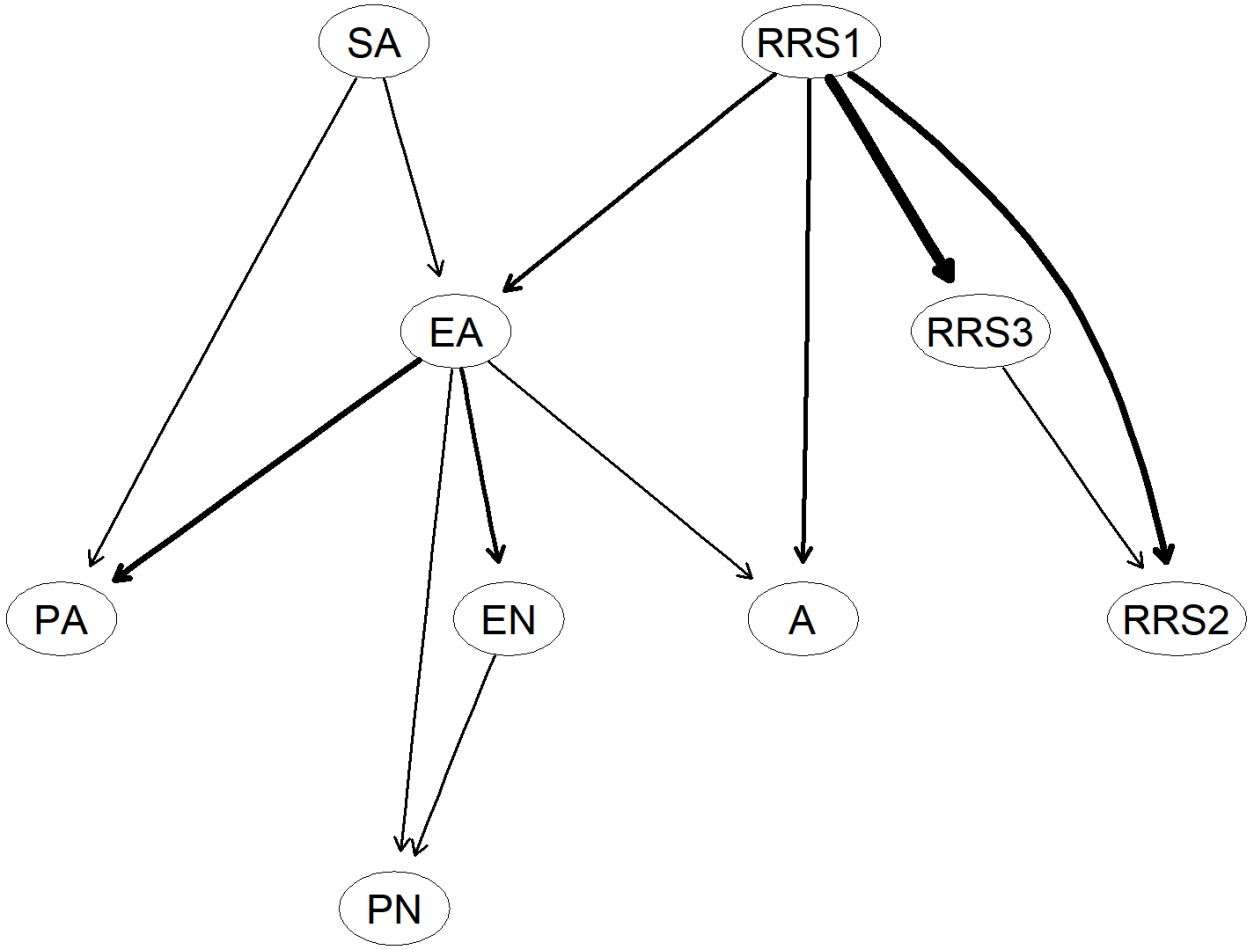
**Bayesian network estimated using the hill-climbing algorithm displayed as a directed acyclic graph (DAG)**. Symptoms are represented as nodes, and edges indicate directional relationships between nodes. The thickness of each edge reflects the magnitude of directional influence—the thicker the edge, the stronger the effect size. Arrow directions represent the direction of conditional dependency.

Fig. 4 illustrates the Bayesian network depicting the relationships among adolescent self-harm addiction, trauma, and rumination. The topological structure of the DAG helps distinguish upstream from downstream variables, providing a basis for exploring potential causal relationships. Symptoms positioned at the top of the DAG are considered to have greater predictive priority and clinical significance. Emotional abuse appeared at the highest level of the network, implying causal priority and a higher probability of triggering symptoms like addictive NSSI and rumination. It may further exacerbate addictive tendencies. Bayesian Information Criterion (BIC): A statistical criterion for model selection that balances goodness-of-fit with model parsimony by penalizing complexity; a lower value indicates a more optimal and stable model structure. The network’s BIC score (–29,046.08) confirmed its stability.

#### 3.2.4 Network Comparison Test

**Fig. 5.**
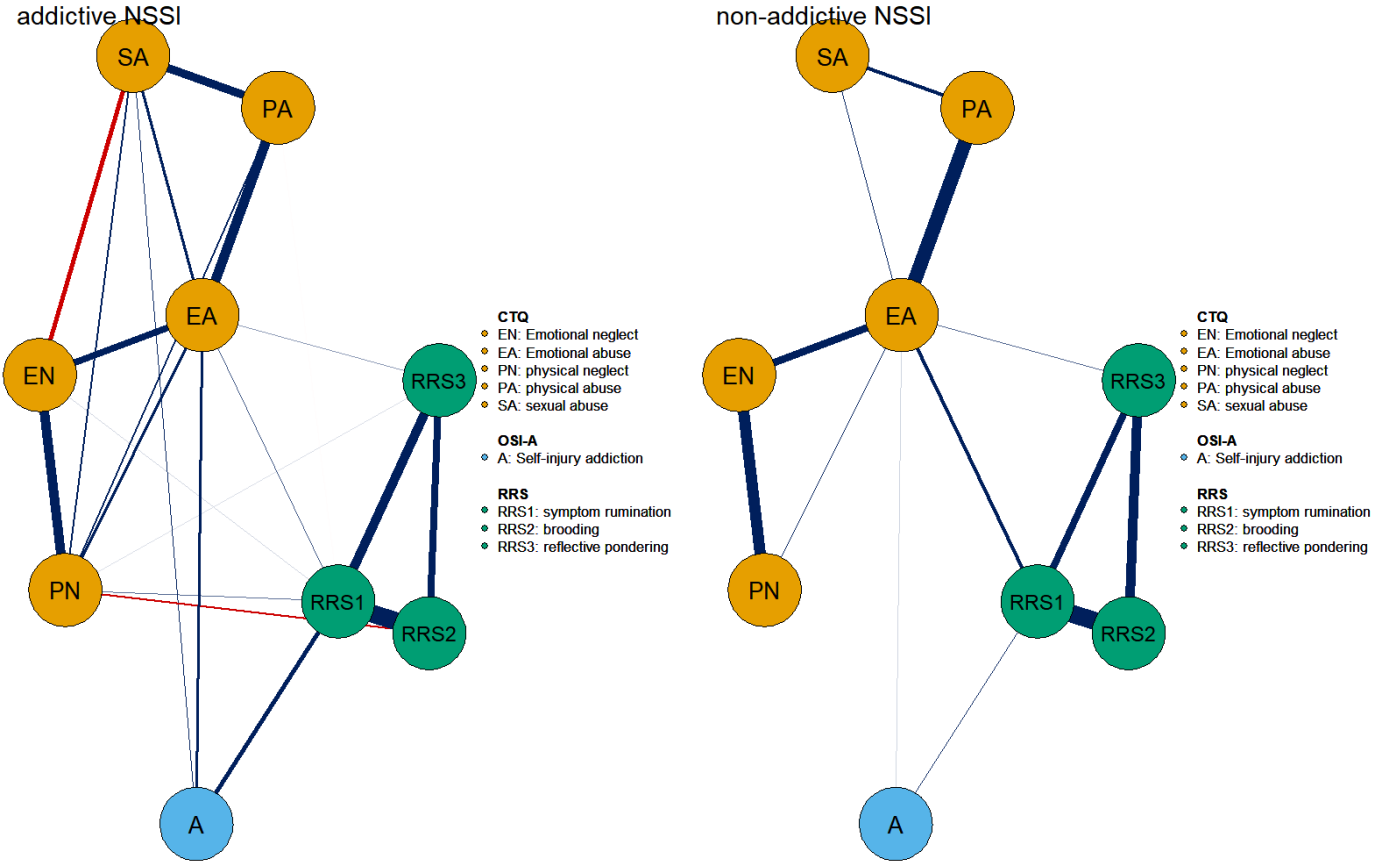
**Network comparison between addictive NSSI (left) and non-addictive NSSI (right)**. In the networks, blue nodes represent self-injury addiction, green nodes denote rumination, and orange nodes indicate trauma. Blue edges correspond to positive correlations, red edges to negative correlations, and edge thickness reflects the strength of the associations between nodes.

As shown in **Supplementary Fig. 4**, permutation-based NCT analyses indicated that addictive and non-addictive NSSI networks did not differ significantly, either in structure invariance (max edge-weight difference = 0.19, *p* = 0.11) or in global strength (difference = 1.06, *p* = 0.06). Moreover, 97.22% of the edges (35/36) showed no significant differences (*p* > 0.05). The addictive group showed a stronger A–RRS1 edge (*p* < 0.05) than the non-addictive group. Networks are shown in Fig. 5.

#### 3.2.5 Comparison of Networks Adjusted for a Covariate

The Spearman correlations between the original and sex-adjusted network models were high across all three networks, indicating minimal structural differences after controlling for gender. Specifically, the correlation coefficients were r = 0.988 (*p* < 0.001) for the overall NSSI network, r = 1.000 (*p* <*0*.001) for the addictive group network, and r = 0.987 (*p* <*0*.001) for the non-addictive group network. These findings suggest that including sex as a covariate did not substantially alter the network structure.

#### 3.2.6 Mediation Analysis

**Fig. 6.**
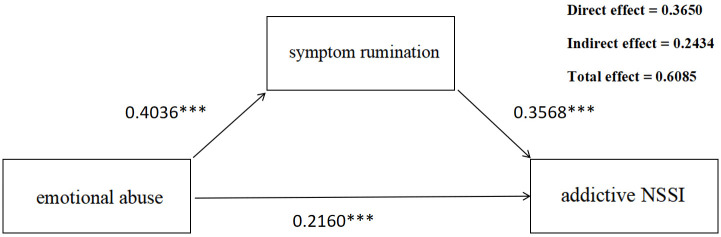
**Mediation Analysis of the Effect of Symptom rumination on addictive NSSI via emotional abuse**. ***p < 0.001.

Based on the results and structure derived from both undirected and Bayesian networks, several key nodes were incorporated into the structural equation modeling. As shown in Table 3 and Fig. 6, emotional abuse exerted not only a direct effect on addictive NSSI but also an indirect effect through symptom rumination, indicating a mediation pathway. Specifically, the direct effect (0.3650) and indirect effect (0.2423) accounted for 59.98% and 40.00% of the total effect (0.6085), respectively.

**Table 3.** **Mediation analysis results: total, direct, and indirect effects with confidence intervals**.

Effect type	Effect (β)	SE	LLCI (95% CI)	ULCI (95% CI)	Proportion of total effect
Total effect	0.6085	0.0468	0.5167	0.7002	
Direct effect	0.3650	0.0477	0.2715	0.4586	59.98%
Indirect effect	0.2434	0.0253	0.1968	0.2939	40.00%

SE, standard error; LLCI, lower level of confidence interval; ULCI, upper level of confidence interval.

## 4. Discussion

While this study was originally guided by the I-PACE model [15], our findings allow us to further propose a Network Intervention Model for NSSI Addiction. This model highlights emotional abuse as the core upstream node and symptom rumination as the key mediating pathway, thereby providing a more targeted theoretical framework for intervention. To further test the directional associations indicated by the DAG, we built a mediation model in which rumination served as the mediator linking emotional abuse to addictive NSSI. By clarifying these central nodes and pathways, the proposed model extends I-PACE and offers novel contributions by linking trauma-related adversity with cognitive processes in addictive self-injury and suggesting targeted interventions such as rumination-blocking therapies. While the network comparison revealed no significant group differences in overall structure or global strength, the connection between NSSI addiction and symptom rumination was notably stronger within the addiction group.

In the undirected trauma–rumination–addiction network constructed in this study, we identified several key bridge nodes facilitating cross-community transmission, namely addictive features, symptom rumination, and emotional abuse. Among these, addictive features exhibited the highest bridge centrality, forming the strongest intra-community connections with symptom rumination and emotional abuse, located respectively at the cores of the rumination and trauma subcommunities. Moreover, the edge connecting symptom rumination and emotional abuse represented the strongest link between the two subcommunities, highlighting its pivotal role in cross-community connectivity. In the DAG, we further delineated potential causal pathways among the three nodes: emotional abuse exerted a direct effect on addictive features, while also contributing indirectly via symptom rumination. This pathway was supported by subsequent mediation analysis, which revealed a significant direct effect of emotional abuse on addictive features, with the indirect effect via symptom rumination accounting for 40.00% of the total effect. In a sample of 833 depressed adolescents in China, rumination was found to significantly mediate the trauma–NSSI relationship [28]. This study further revealed the impact of emotional abuse and symptom rumination on addictive NSSI. These findings suggest a synergistic interplay between early traumatic experiences and cognitive processing styles in the development of addictive self-injury behaviors. Within the framework of Adolescent Adverse Experiences (AAEs), symptom rumination (as an individual-level cognitive factor) and emotional abuse (as interpersonal-level adversity) jointly amplify addictive self-injurious behavior through cross-level interaction. Specifically, the tendency toward rumination may exacerbate the impact of childhood trauma, thereby intensifying the addictive characteristics of self-injurious behavior [29]. This model explains the ecological interaction between adverse cognitive processing and interpersonal adversity. For individuals with high rumination traits, a history of emotional abuse may enhance the reactivation of trauma-related memories, reinforce persistent negative thought cycles [30], and thereby increase the risk of repetitive and compulsive self-injury behaviors. Adolescents with a strong ruminative style are particularly vulnerable to the effects of trauma, as they tend to fixate on their emotional suffering [31], which in turn may exacerbate the progression toward self-injury addiction. This study elucidates a key pathway linking emotional abuse, symptom rumination, and NSSI addiction, offering a novel network-based perspective on the addiction of NSSI.

Additionally, the findings suggest that targeting bridge nodes may be a more effective overall network intervention strategy compared to focusing on other centrally connected nodes [32]. Rumination refers to a cognitive-emotional process in which individuals persistently dwell on thoughts and emotions related to adverse life events [33]. A large-scale study involving 1782 adolescents with depression found that rumination significantly influenced the frequency of NSSI behaviors, underscoring the importance of cognitive vulnerability in self-injury [34]. Our findings further demonstrate that symptom rumination plays an important role in shaping the addictive features of NSSI. Our comparison indicated that although overall network structures were similar across groups, the addictive group demonstrated a significantly stronger edge linking rumination with NSSI addiction. As such, by removing or disrupting the connection between “symptom rumination”, “emotional abuse” and “self-injury addiction”, we may begin to deactivate the network of trauma, rumination, and addiction and alleviate the adverse effects of addictive NSSI. While existing NSSI interventions have mainly emphasized restructuring maladaptive thought content, the present findings underscore the importance of targeting cognitive processes, particularly symptom rumination associated with emotional abuse. Building on the network structure identified in this study, we propose a stepwise intervention framework: (1) systematic screening for emotional abuse using validated assessment tools; (2) stratification of adolescents into distinct risk profiles; and (3) implementation of targeted rumination-blocking interventions (e.g., rumination-focused cognitive behaviour therapy or complementary approaches such as music therapy [35, 36]). Such a structured framework may enhance both the translational value and practical applicability of prevention and treatment strategies for adolescent NSSI addiction.

Despite its contributions, the present study is not without limitations. First, participants were recruited from an outpatient psychiatric population, with a predominance of female respondents. Although we conducted network comparison tests between the addiction and non-addiction subgroups, the results revealed no significant differences in network structure. This may be attributable to sampling bias due to the overrepresentation of female participants. Future studies should strive for more gender-balanced samples to mitigate potential biases and enhance generalizability. The use of convenience sampling from a single tertiary psychiatric hospital and an online platform may introduce selection bias and limit the generalizability of our findings. Community-based adolescents may present different network characteristics; thus, future studies should recruit from community populations to examine whether the results extend beyond clinical settings. Second, the cross-sectional design of this study precludes definitive conclusions about causality. Although a DAG approach was applied to infer plausible directional associations, these should be interpreted with caution. The positioning of emotional abuse upstream in the DAG suggests a potential priority for further exploration, but longitudinal research is required to establish temporal ordering and causality more robustly. While we aggregated items into dimension-level nodes to enhance interpretability and model stability, this approach may obscure heterogeneity at the item level. Future studies with larger samples and longitudinal designs are warranted to replicate and refine these findings. Third, regarding the construct of rumination, previous research has emphasized that it encompasses not only cognitive content but also motivational, behavioral, and metacognitive components [37]. We acknowledge that our operationalization did not capture the full breadth of these dimensions. Rather, we relied on widely used instruments that reflect only specific facets of rumination. While this approach enhances comparability with prior studies, it limits the comprehensiveness of our assessment. Future research should aim to refine theoretical definitions and develop more integrative measurement tools. Moreover, the study did not collect clinical indicators (e.g., clinical diagnosis, illness severity, and treatment status), which may have introduced unmeasured confounding and may limit the interpretability and generalizability of the findings.

This study applied undirected network analysis, Bayesian approaches, and mediation modeling in a clinical adolescent sample to examine the links between NSSI addiction, trauma, and rumination. The findings highlighted key symptoms within the undirected network, identified emotional abuse as an upstream factor through DAG analysis, and further supported this role in the mediation model. It is recommended that future studies further replicate the current research and focus on solving symptom rumination to reduce the impact of emotional abuse on addiction.

## 5. Conclusion

This study demonstrated undirected and directed networks linking trauma, rumination, and NSSI addiction in adolescents. Symptom rumination and emotional abuse emerged as central bridging nodes in the network of addictive self-injury, with exploratory findings highlighting associations that warrant longitudinal investigation to clarify their temporal dynamics. Rumination was more strongly linked to NSSI addiction within the addiction group. These results emphasize the need for early detection and targeted intervention on bridge symptoms—especially trauma-related rumination—to mitigate the onset and persistence of NSSI addiction.

## Availability of Data and Materials

The corresponding author will provide the data that underpin the study’s conclusions with a reasonable application.

## Supplementary Material

Supplementary material associated with this article can be found, in the online version, at https://doi.org/10.31083/AP44340.
