# Supplementary material for: Interrelationships Between Self-Injury Addiction, Traumatic Experiences, and Rumination Among Adolescents With Non-Suicidal Self-Injury: A Network Analysis
Source: Alpha Psychiatry. 2026 Feb 3;27(1):44340. doi: 10.31083/AP44340 (PMC12957973; doi:10.31083/AP44340)

**Supplemental materials**

**Figure legends**

**Supplementary Fig. 1.** Bootstrapped difference test for edge weight of trauma, rumination and self-injury addiction self-injury addiction network.

**Supplementary Fig. 2.** Bootstrapped test for confidence intervals of edge, EI and BEI of trauma, rumination and self-injury addiction network.

**Supplementary Fig. 3.** Bootstrapped difference test for expected influence of trauma, rumination and self-injury addiction network.

**Supplementary Fig. 4.** Network comparison test between addicted and non-addicted groups.

**Supplementary Table 1.** Edge weight matrix of the trauma, rumination and self-injury addiction network.

|  | A | EN | EA | PN | PA | SA | RRS1 | RRS2 | RRS3 |
| --- | --- | --- | --- | --- | --- | --- | --- | --- | --- |
| A | 0.00 |  |  |  |  |  |  |  |  |
| EN | 0.00 | 0.00 |  |  |  |  |  |  |  |
| EA | 0.13 | 0.29 | 0.00 |  |  |  |  |  |  |
| PN | 0.04 | 0.35 | 0.12 | 0.00 |  |  |  |  |  |
| PA | 0.01 | -0.01 | 0.41 | 0.09 | 0.00 |  |  |  |  |
| SA | 0.08 | -0.15 | 0.14 | 0.11 | 0.28 | 0.00 |  |  |  |
| RRS1 | 0.23 | 0.07 | 0.11 | 0.09 | -0.03 | -0.05 | 0.00 |  |  |
| RRS2 | 0.00 | -0.08 | 0.03 | -0.11 | 0.00 | -0.01 | 0.59 | 0.00 |  |
| RRS3 | 0.00 | 0.00 | 0.06 | 0.05 | 0.00 | 0.04 | 0.29 | 0.30 | 0.00 |

**Supplementary Table 2.** Basic information and network inference of items used to self-injury addiction, trauma and rumination (N=1,169)

| Nodes | Label | **EI** | **BEI** |
| --- | --- | --- | --- |
| **OSI-A** | | | |
| self harm addiction | A | -0.09 | 1.58 |
| **CTQ** | | | |
| Emotional neglect | EN | -1.07 | -0.71 |
| Emotional abuse | EA | 1.54 | 0.83 |
| physical neglect | PN | -0.43 | -0.39 |
| physical abuse | PA | -0.27 | -0.73 |
| sexual abuse | SA | -1.56 | -0.36 |
| **RRS** | | | |
| symptom rumination | RRS1 | 1.20 | 1.21 |
| brooding | RRS2 | 0.11 | -1.43 |
| reflective pondering | RRS3 | 0.57 | 0.01 |

**Supplementary Fig. 1.** Bootstrapped test for confidence intervals of edge, EI and BEI of trauma, rumination and self-injury addiction network


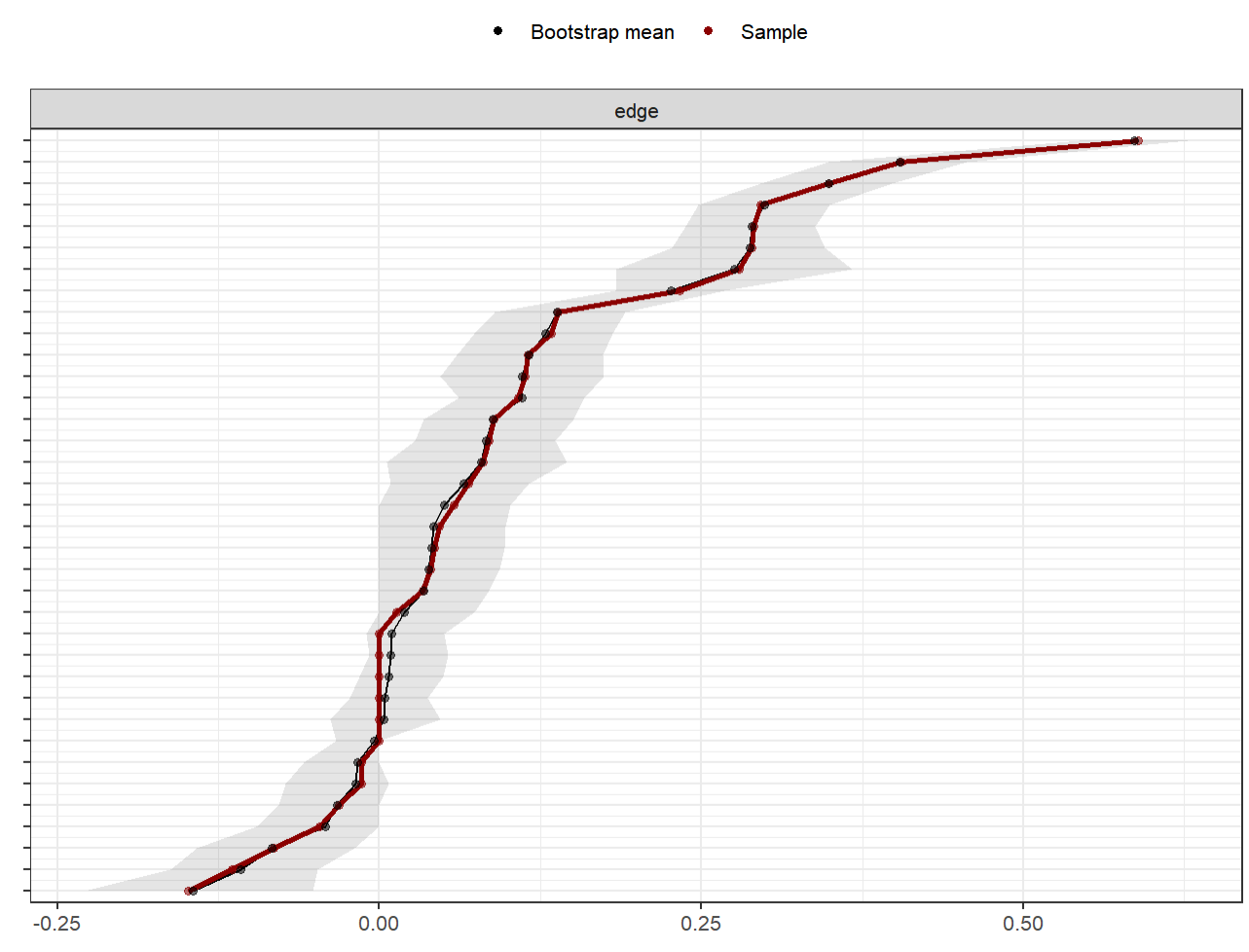


**Supplementary Fig. 2.** Bootstrapped difference test for edge weight of trauma, rumination and self-injury addiction self-injury addiction network


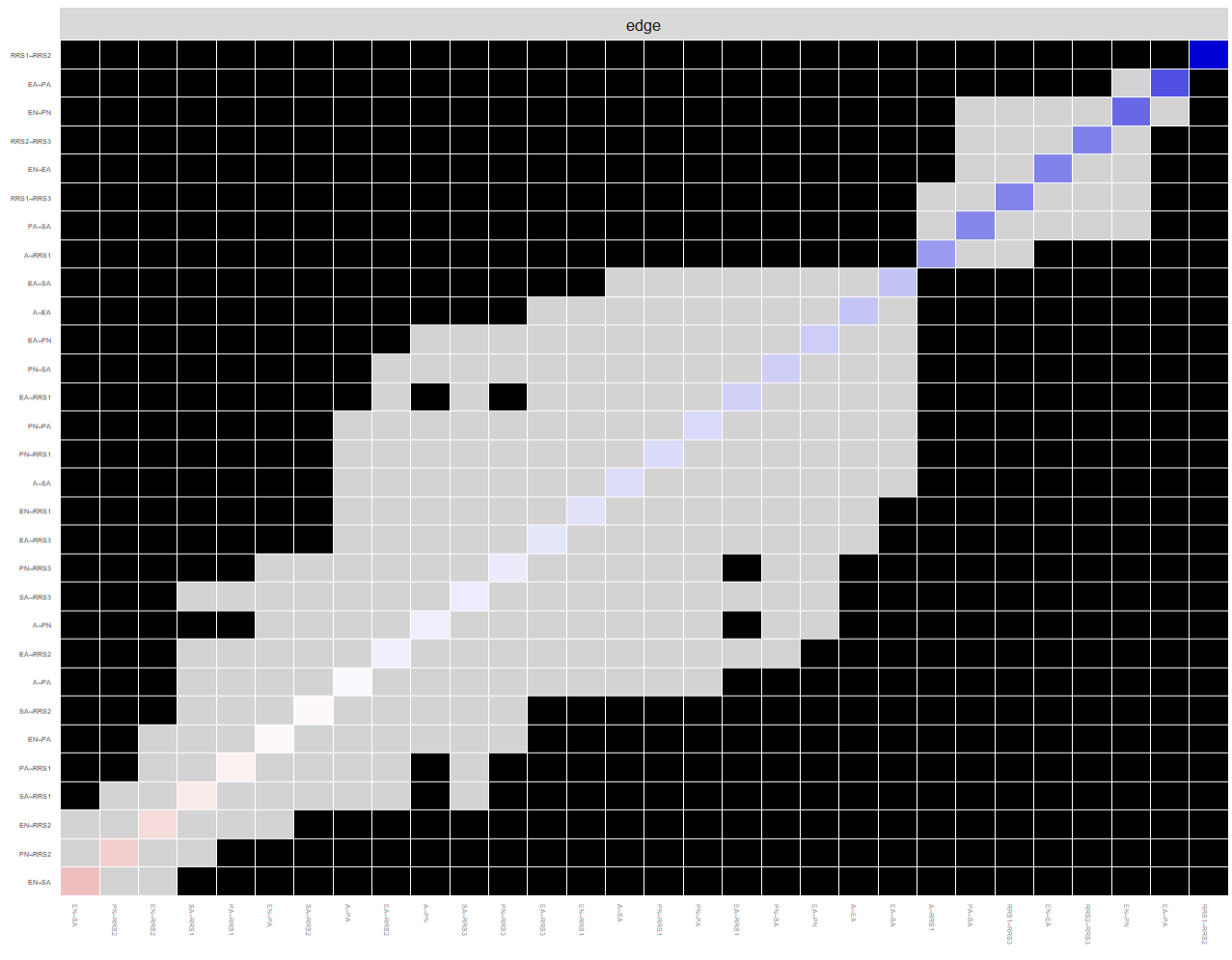


**Supplementary Fig. 3.** Bootstrapped difference test for expected influence of trauma, rumination and self-injury addiction network


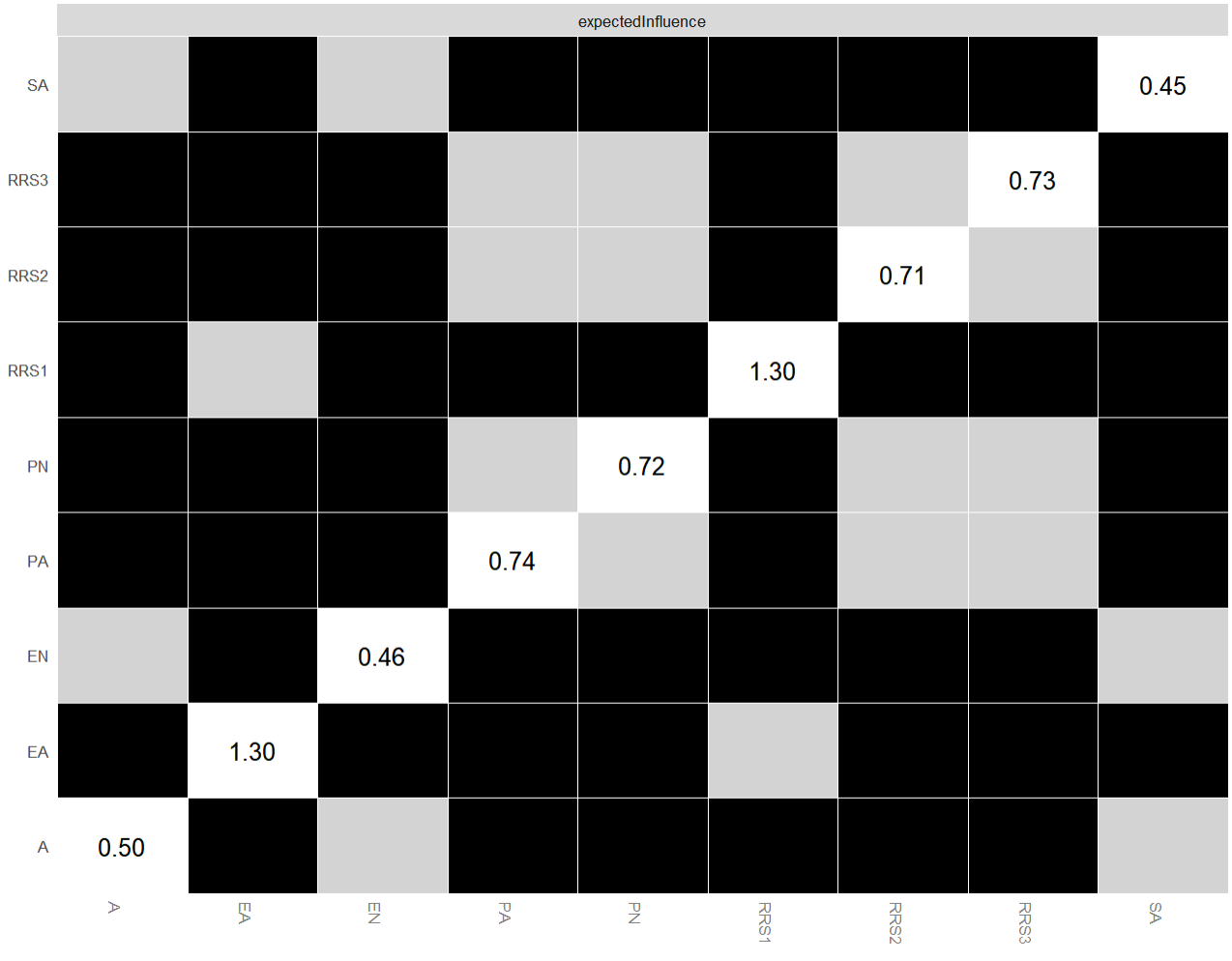


**Supplementary Fig. 4.** Network comparison test between addicted and non-addicted groups.


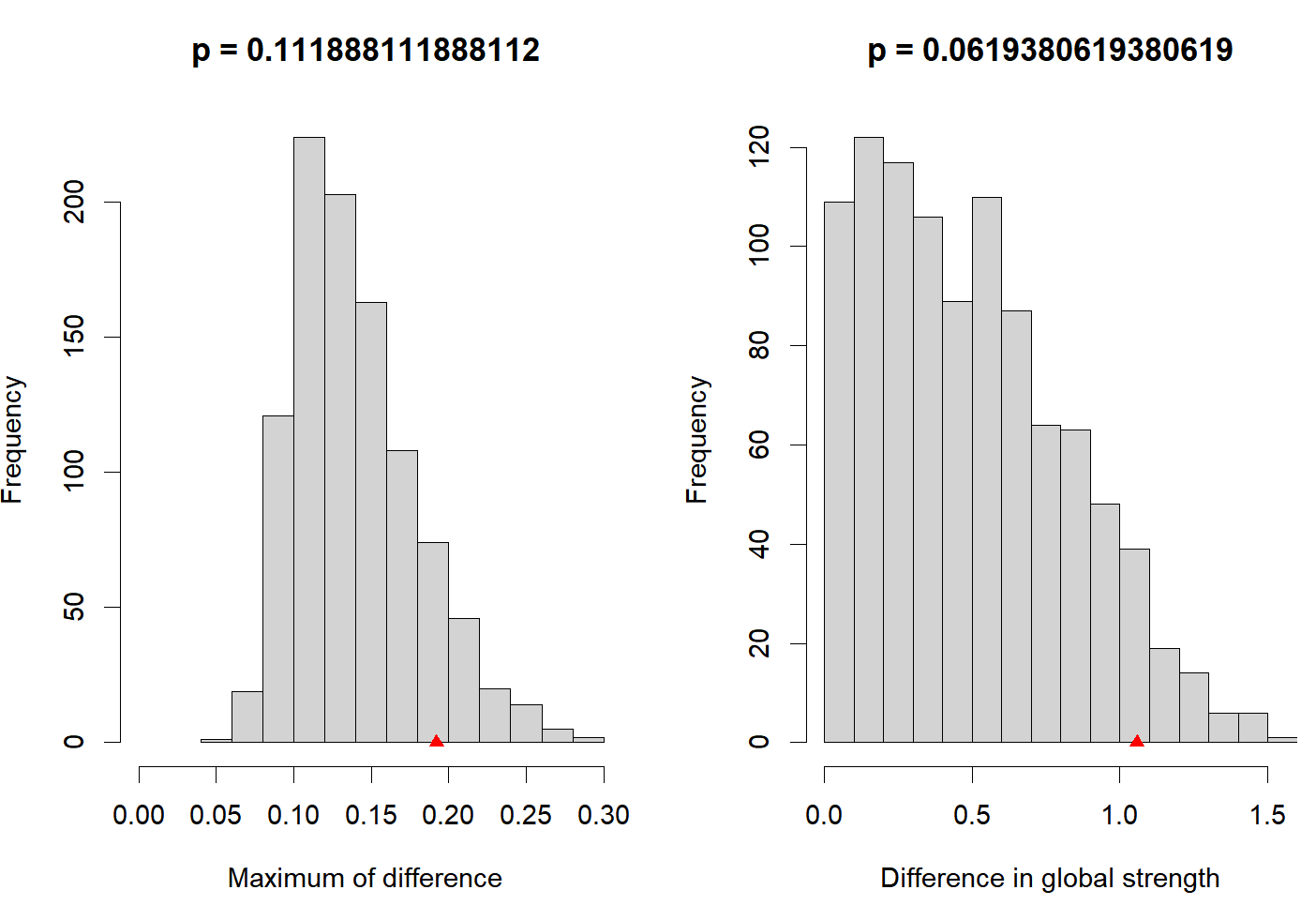

Supplement: Supplementary file 1 [file 2757-8038-27-1-44340-s1.zip › Supplementary Material.docx]
